# Haitian Mamas: The Development and Two-Year Implementation of an OB/GYN-Based Preventive Behavioral Health Group in a Safety-Net Hospital Setting

**DOI:** 10.3390/bs16101951

**Published:** 2026-10-20

**Authors:** Kalo C. Sokoto, Johanna Milord, Joelle T. Taknint, Resham Gellatly, Audrey Montgomery, Maithri Amesekere, Kettie Louis, Layal Al Mosawi, Muthoni Muthiga, David C. Henderson

**Affiliations:** 1 Department of Psychiatry, Boston Medical Center, Boston, MA 02119, USA; 2 Department of Counseling Psychology and Applied Human Development, Boston University, Boston, MA 02215, USA; 3 Department of Counseling Psychology, University of Wisconsin-Madison, Madison, WI 53706, USA; taknint@wisc.edu; 4 Massachusetts General Hospital, Boston, MA 02114, USA; 5 Boston Medical Center, Boston, MA 02119, USA; 6 Department of Psychiatry, Chobanian & Avedisian School of Medicine, Boston University, Boston, MA 02118, USA; 7 Department of Obstetrics and Gynecology, Chobanian & Avedisian School of Medicine, Boston University, Boston, MA 02118, USA; 8 Dr. Layal Al Mosawi Psychological Services, New York, NY 10011, USA; 9 Department of Psychiatry, Stellenbosch University, Cape Town 7505, Western Cape, South Africa

**Keywords:** perinatal mental health, integrated behavioral health, group therapy, forced migration

## Abstract

Perinatal mental health conditions are among the leading causes of maternal mortality in the United States. However, 50% to 70% of perinatal mental health cases go undiagnosed, and of those diagnosed, fewer than 30% receive treatment. At Boston Medical Center, a large safety-net academic medical center in the northeastern United States (US), this treatment gap became apparent when new Refugee Women’s Health Clinic patient visits increased by 138% following an influx of Haitian forced migrants starting in 2021. In response, we developed and implemented Haitian Mamas, a same-day access preventive behavioral health group within the Obstetrics and Gynecology department. This quality improvement study evaluates the development of Haitian Mamas from August 2023 to 2025, using four Plan–Do–Study–Act (PDSA) cycles. We evaluated the feasibility of this program across six domains: reach, engagement, integration of measurement-based care, financial viability, adaptability, and provider-perceived acceptability. Over two years, 196 women participated in the group, with an average of eight members per group, and there was a progressive improvement in integrating measurement-based care, which rose from 11.5% in Cycle 1 to 67.4% in Cycle 4. 35% screened positive for moderate to server mental health concerns. A thematic analysis of qualitative data from providers’ weekly post-group reflections identified implementation barriers and facilitators for Haitian Mamas. Our findings suggest that the development and implementation of a same-day access, OBGYN-based preventive behavioral health group for perinatal patients in a safety-net hospital setting is operationally feasible.

## 1. Introduction

Haiti’s 2021 political crisis led to rapid forced displacement: by 2023, Massachusetts was sheltering 882 migrant families in hotels, up from less than 10 in 2021 (Damiano, 2023). Boston Medical Center, a large safety-net academic medical center in the northeastern US, felt the impact of this crisis at the hospital’s Refugee Women’s Health Clinic (RWHC), a subspecialty clinic within Obstetrics and Gynecology (OB/GYN). New patient visits surged from 170 to 405 between 2022 and 2024, with 91% of patients identifying Haitian Creole as their primary language, stretching the existing OB/GYN staffing and behavioral health service capacity (Taknint et al., 2026). At the time, Boston Medical Center’s OB/GYN behavioral health care was referral-based and individually offered, and no same-day access, group-based, linguistically concordant behavioral health service existed within OB/GYN.

### 1.1. Background

Perinatal mental health is among the leading causes of maternal mortality in the US (World Health Organization, 2024). However, 50% to 70% of perinatal mental health (PMH) conditions go undiagnosed (Cox et al., 2016). Migrant pregnant women, including forced migrants, are disproportionately affected by PMH conditions (Anderson et al., 2017; Laumonnier et al., 2026; Stevenson et al., 2023). In this manuscript, we use the term forced migrants to refer to people who have left their home countries because of conflict, persecution, violence, or disaster, regardless of legal status (Castles, 2003). These women also have low rates of behavioral health (BH) utilization (Derr, 2016), potentially due to language barriers, isolation, and lack of childcare (Markey et al., 2024; Webb et al., 2023). Furthermore, BH referral pathways are typically individual-based, require pre-diagnostic evaluation, and are located away from the OB/GYN clinic (Carroll et al., 2020; Yeshitila et al., 2024).

OB/GYN providers also have limited capacity and time to screen and refer (Poleshuck & Woods, 2014), while standard screeners are often untranslated, unvalidated, and inattentive to trauma symptoms (Itani et al., 2025). Providers serving perinatal forced migrant patients face resource limitations, including language and cultural barriers, and institutional policies not designed for this population (Suphanchaimat et al., 2015). For example, when patients express distress that does not meet a diagnostic threshold, providers can be left feeling helpless to act, a persistent experience identified as moral distress (Middlemiss, 2024; Vinckers & Landeweer, 2025). Integrated behavioral health (IBH) care models have been used to increase access to behavioral health in specialty clinics, including in OB/GYN (Keedle et al., 2023; McNeil et al., 2024; Myors et al., 2013). Preventive, low-intensity behavioral health interventions embedded within OB/GYN can help address patient engagement with BH and, in turn, providers’ moral distress.

### 1.2. Rationale

At Boston Medical Center, a structured needs assessment (January–May 2023) confirmed that no preventive, same-day access, culturally tailored behavioral health support existed for Haitian perinatal women (Taknint et al., 2026). This gap laid the foundation for the development of a stratified Refugee Women’s Health Clinic Behavioral Health (RWHC BH) Care Model embedded in the department of OB/GYN (Ijebor et al., 2026; Taknint et al., 2026). The Haitian Mamas group, the focus of this manuscript, served as both the preventive and step-down care arm of the RWHC BH model.

Three frameworks informed the Haitian Mamas group, each operationalized through specific program components: the IWBH framework (Crawford et al., 2022) posits that women’s health settings, such as OB/GYN, are key to wellness-centered behavioral health care; the six trauma-informed care (TIC) principles from the Substance Abuse and Mental Health Services Administration (SAMHSA, 2014) shaped the group’s clinical content and patient engagement; and the PDSA cycle (Langley et al., 2009) enabled iterative development based on observed needs. Together, these frameworks provided practical tools for addressing the behavioral health needs of perinatal forced migrant women, reflected in the group’s design.

### 1.3. Specific Aims

This quality improvement study had two objectives: (1) to develop a same-day access, OB/GYN-based behavioral health service delivery model in a safety-net hospital setting using a PDSA approach and (2) to determine the feasibility of this delivery model for perinatal forced migrant women. We assessed feasibility across six domains: reach, engagement, integration of measurement-based care, financial viability, adaptability, and provider-perceived acceptability.

## 2. Materials and Methods

### 2.1. Context

Boston Medical Center is a safety-net academic medical center serving one of the most linguistically and socially complex obstetric populations in the northeastern United States. The OB/GYN department delivers approximately 3200 babies annually. The majority of patients identify as Black, Indigenous, or people of color (87%) and carry public insurance (80.2%). The most common languages spoken among patients are English, Haitian Creole, and Spanish. Per hospital policy, we do not collect patient immigration status; therefore, it is not available for analysis. Appendix A provides the demographics of perinatal Haitian Creole-speaking patients receiving obstetrical care at Boston Medical Center during the study period (2023–2025; n = 1425).

The Refugee Women’s Health Clinic (RWHC) is an interdepartmental collaboration between Psychiatry, Internal Medicine, and OB/GYN, providing comprehensive, culturally sensitive women’s health services to refugee, asylum-seeking, and immigrant communities. At the time of this quality improvement initiative, existing group-based programs focused on prenatal care; however, they did not address behavioral health needs, leaving patients dependent on external referrals to outpatient mental health services, a process associated with barriers to behavioral health access (Markey et al., 2024; Webb et al., 2023).

### 2.2. Intervention Description

The Haitian Mamas group was developed as a quality improvement initiative using the Plan–Do–Study–Act (PDSA) framework (Langley et al., 2009). Unlike frameworks that evaluate the implementation of an existing intervention, Haitian Mamas was built iteratively. The intervention described below reflects the group in its present form using the TIDieR (Template for Intervention Description and Replication; Hoffmann et al., 2014) checklist and guide, sections one to eight.

#### 2.2.1. Name

The Haitian Mamas (HM) group met in person weekly for one hour at the RWHC in the OB/GYN department at Boston Medical Center.

#### 2.2.2. Why

The goal of the group was to provide accessible and linguistically responsive behavioral health care. In line with this goal, Haitian Mamas served as both the preventive and step-down care arms of the RWHC BH Care Model.

#### 2.2.3. What (Materials)

##### Assessment

The Refugee Health Screener-15 (RHS-15) is a validated 15-item measure assessing the most common mental health symptoms amongst forcibly displaced people.

##### Food

A Haitian caterer provided a hot meal for mothers at every session, while case managers provided nonperishable food items. This addressed the food insecurity many participants faced due to living in shelters.

##### Child Minding

Group members were welcome to bring their young children, and medical student volunteers provided child minding in an adjacent room.

##### Case Management Resources

The group provided donated diapers and clothing to members when possible. The group consistently provided community resource lists, including legal and immigration, housing, transportation, and childcare services.

##### Funding

The group received partial financial support through a Refugee Mental Health Initiative (ReMHI) Grant from the Massachusetts Department of Public Health Department of Public Health.

#### 2.2.4. What (Procedures)

##### Referral Pathway

OB/GYN and behavioral health providers made referrals through warm hand-offs to the behavioral health team or through the Electronic Health Record system. Self-referrals and walk-ins were accepted. Eligibility criteria included identifying as Haitian, speaking Haitian Creole, and being a perinatal patient receiving care in the OB/GYN department. No pre-diagnostic evaluation or psychiatric diagnosis was required.

##### Session Structure

Facilitators completed a six-session perinatal mental health training series for RWHC BH clinicians in July and August 2023 as the program launched, with each session lasting one hour. The series included three sessions on the National Curriculum in Reproductive Psychiatry, with adaptations for RWHC patients, and one session each on prescribing psychopharmacology during pregnancy, treating post-traumatic stress disorder (PTSD) during pregnancy, and culturally responsive care. Facilitators also received weekly supervision from licensed psychologists with expertise in immigrant and perinatal mental health. There was no fixed curriculum for Haitian Mamas. Content was emergent and responsive to participant needs (Elwy et al., 2020), with particular emphasis on psychoeducation and coping skills related to reported trauma, mood, and anxiety symptoms.

##### Graduation

After completing five sessions, or upon request, each participant received an individual graduation ceremony, including a certificate and a small gift of baby supplies. During graduation, group members were invited to reflect on their HM experience, guided by the following questions: (1) How did you learn about Haitian Mamas? (2) What kept you coming? (3) What did you get out of participating? (4) What recommendations do you have? Facilitators recorded de-identified summaries of responses in field notes.

#### 2.2.5. Who Provided 

There was an interdisciplinary team collaborating to offer the Haitian Mamas. The role, qualifications, and function of each provider involved is described below (See Table 1).

#### 2.2.6. How

##### Session Delivery

Sessions were delivered in person, in a group format, mediated by an in-person Haitian Creole interpreter (most co-facilitators did not speak Haitian Creole). Group size was capped at 12 participants per session: eight prescheduled and four reserved for same-day access participants identified by OB/GYN providers during that day’s clinic.

##### Where

Sessions were held in a group space within the OB/GYN department at Boston Medical Center. The group was held in the same location and often on the same day as participants’ obstetric appointments.

##### When and How Much

Sessions were held once per week for 60 min. Participants could enroll at any point during the perinatal period, defined as pregnancy through the first year postpartum, join at any session, and attend without a waiting list. Five sessions of attendance signaled graduation.

### 2.3. Evaluative Approach

We evaluated feasibility across the six domains using clinically meaningful, observable indicators in a real-world embedded setting (Bowen et al., 2009; Proctor et al., 2011). We set benchmarks prospectively and retrospectively, depending on whether an institutional standard existed. Where none existed, we assessed feasibility retrospectively, consistent with quality improvement projects (Bellin & Dubler, 2001). We did not administer validated implementation outcome measures. Haitian Mamas was built as a clinical program first, and its content emerged and responded to participant needs (Weiner et al., 2017). Adding surveys would also have increased burden for patients and staff in a same-day access clinical setting.

We evaluated reach descriptively through enrollment, with no prospective target. We benchmarked engagement against the institution’s minimum session-utilization threshold of four participants per session and set no individual dose target, given the group’s open access design (Elwy et al., 2020). We assessed behavioral health screening integration (RHS-15) via administration rate across PDSA cycles, with a 100% prospective target. We contextualized screening rates against overall Boston Medical Center’s perinatal screening rates during the same period (Baul et al., 2026) and defined financial viability narrowly as whether group billing could offset clinician time costs. Behavioral health leadership set a prospective benchmark, at least four billable patients per session, the minimum needed to match Medicaid reimbursement for one hour of individual psychotherapy. We assessed adaptability by tracking structural changes across the four PDSA cycles (Table 2) and their association with subsequent engagement, screening, and provider-reported outcomes, with no prospective adaptation targets. We assessed provider-perceived acceptability retrospectively through reflexive thematic analysis of field notes, with no prospective benchmark. Patient-perceived acceptability is reported separately (Sokoto et al., under review).

### 2.4. Measures

#### 2.4.1. Field Notes

We collected data between August 2023 and August 2025. Provider content included providers’ reflections on their experience, group adaptations, and group content, documented immediately after each group session. Over these two years, four providers contributed 74 field notes, writing a few sentences to several paragraphs after most group sessions, with at least one volunteer and the program coordinator also helping record patient feedback. The field notes were collected in a confidential, shared Word document. From the start of the program in August 2023, all providers used the same three guiding prompts: What is working? What is not working? What changes are needed? Providers responded to these prompts in free text.

#### 2.4.2. Quantitative Data

The Refugee Health Screener-15 (RHS-15) was used to screen patients before or at the first session of Haitian Mamas and at graduation. The RHS-15 is a validated 15-item measure assessing the most common mental health symptoms amongst forcibly displaced people, including depression, anxiety, post-traumatic stress (PTSD), general distress, and coping (Hollifield et al., 2013). The RHS-15, which has been translated and validated in Haitian Creole, has strong sensitivity/specificity for depression (0.95/0.89), anxiety (0.94/0.86), and post-traumatic stress disorder (PTSD; 0.81/0.87) (Hollifield et al., 2013). The RHS-15 has been supported for use in obstetrical settings (Johnson-Agbakwu et al., 2014; Soldati et al., 2025), is validated for identifying perinatal depression (Fellmeth et al., 2018), and can reliably detect symptom change (Taknint et al., under review). Notably, the RHS-15 served only as a screening and symptom-monitoring tool. Within the clinical workflow, any positive RHS-15 screen triggered a referral for a full diagnostic psychiatric evaluation. The measurement goal was to administer the Refugee Health Screener-15 (RHS-15) at patients’ first session and again at graduation if they reached the five-session cap. Group leaders reviewed screening data and used them to guide referrals.

### 2.5. Analysis

#### 2.5.1. Quantitative Data Analysis

Descriptive statistics characterized the distribution of scores across the sample. We calculated 95% confidence intervals (CIs) for key proportions using the Wilson score method and defined positive screens as a score ≥ 12 on the first 14 items or a distress thermometer score ≥ 5. Health care settings, particularly resource-constrained ones, may need to tailor screening cutpoints to use the best available resources (Hollifield et al., 2016). Accordingly, we also analyzed symptom severity levels, which differ from the positive/negative screen scoring method, using the cutpoints established by Bjärtå et al. (2018). Differences in attendance rates between participants who were and were not screened were assessed using Welch’s *t*-test.

#### 2.5.2. Qualitative Data Analysis

Qualitative data were analyzed through reflexive thematic analysis (Braun & Clarke, 2006), adopting a contextualist epistemological position. Field notes were treated as meaningful accounts of real events and observations within the group. At the same time, the themes generated from them were understood as being constructed through the analysts’ interpretive lens rather than as objective, singular truths independent of that lens. This approach was selected for its suitability to datasets generated by participant-practitioners reflecting on a real-world clinical intervention over time. The two first authors carried out the analysis in six phases: (1) both reviewed all field notes for initial impressions, (2) coded pages 1–13 independently for initial calibration, (3) paused to realign on the research question, and then (4) ran a second calibration round on the next 17 pages, tracking each code by page number for traceability (see Appendix B). The analysts documented themes that both indicated for a specific page section and considered all overlapping areas. For step 5, the two analysts met over several weeks to jointly finalize themes in a dated Excel comparison table (see Appendix C), which also allowed them to track theme frequency over time. The team determined theme saturation through a color-coding process and ongoing dialogue to reach consensus. The team selected quotes presented in the findings directly from the coded pages.

Authors 3 and 4 served as independent auditors (see Section 2.5.4 for their qualifications and the audit process), and the two analysts used reflexive journaling to track how that feedback shaped their decisions. We produced and stored about five dated versions of the coding spreadsheet on secure institutional drives, available on request for auditing. Taken together, PDSA and reflexive thematic analysis complemented each other: PDSA data show what changed structurally and when, while the thematic analysis shows how and why facilitators experienced those changes.

#### 2.5.3. Reflexivity Statement

Two of the four primary data analysts were the group leaders. They also identify as Black women: one of Haitian background and one of Kenyan background. The authors were aware that their identity and professional roles could shape how they processed the data. As group leaders, the lead analysts’ sustained investment in the iterative development of the group shaped their attentiveness to granular, process-level detail in the field notes, likely contributing to the depth and specificity of the operational and adaptive themes (e.g., Group Development and Adaptations, Challenges Arising for Providers) that emerged from the analysis. To reduce the potential bias of serving in dual roles, they used several concrete strategies: analyzing the data with several months of distance between when it was originally written (2023) and when it was analyzed in detail (November 2025–January 2026); attempting to anonymize quotes from the outset by having providers record their observations in a shared documentation system; ongoing memoing; continuous discussion of overlapping identities such as motherhood or shared ethnic background with group members; and consulting external auditors when the data were unclear. Even so, the authors recognize that they are not forced migrants and do not share the same social determinants of health as participants. Ongoing verbal and written reflection helped surface potential bias and strengthen the qualitative rigor of the analysis. See Section 4.4 for a further discussion of dual-role constraints.

#### 2.5.4. Trustworthiness

Two independent auditors, occupying different cultural and professional positions relative to the data, reviewed the coded data and proposed theme structures, providing written feedback on a structured coding document. The auditors were experts in qualitative research, immigrant mental health, and perinatal mental health, and also served as supervisors of the clinical intervention. The team resolved disagreements through consensus: they met to discuss auditor feedback, the lead authors consolidated it, and all co-authors completed a final confirmatory process. Examples of themes and subordinate themes before and after the peer expert review are included in Appendix D. The auditors prompted changes at the categorical and nominal levels, such as changing a verbose subordinate theme title, “negative feelings related to challenging patient’s experience,” to the more focused “moral distress and shared grief,” or dividing the content of a quote between two separate subordinate themes. Analysts and auditors treated disagreements as prompts to examine differing interpretive standpoints. This approach aligns with reflexive thematic analysis’s rejection of coding reliability as a marker of qualitative rigor (Braun & Clarke, 2006). This ongoing peer debriefing and review supported data dependability.

The analysis produced five themes, each with associated subordinate themes, reported in Section 3.2. The team coded manually in Excel and stored the data on a secured Boston Medical Center’sshared drive (see Appendix B and Appendix C).

### 2.6. Ethical Considerations

This project was undertaken as a quality improvement initiative at Boston Medical Center and, as such, was not formally supervised by the Institutional Review Board per their policies. Data derived from de-identified field notes were generated during routine clinical encounters as part of an ongoing quality improvement initiative, rather than collected for the specific purpose of research. We analyzed field notes throughout the project as part of the iterative quality improvement process. We began qualitative analysis of provider perspectives in November 2025 and concluded in January 2026. Quotations were anonymized. Data derived from the Electronic Health Record received an exempt determination (Category 4) from the Institutional Review Board of Boston Medical Center (IRB Number: H-46324; Date of Action: 15 October 2025). A waiver of HIPAA Authorization for Research was approved per 45 CFR 164.512(i)(2)(ii). All procedures in this study followed the ethical standards of the institutional research committee and the 1964 Declaration of Helsinki and its later amendments.

Participation in Haitian Mamas was voluntary at every session. Group members were welcome to attend and were never required to speak, with facilitators reminding them of confidentiality at each session. The co-facilitation model structured the team’s response to participant distress. When a participant became distressed during a session, one co-facilitator provided individual support while the other continued to lead the group. Participants with a positive RHS-15 screen were offered referral for a full diagnostic psychiatric evaluation and higher-intensity care within the RWHC BH Care Model.

## 3. Results

This section describes the results, including participant characteristics and quantitative and qualitative findings.

### 3.1. Program Reach and Participant Characteristics

Across the evaluation period (August 2023–August 2025; 24 months), 196 women participated in Haitian Mamas. Participants ranged in age from 19 to 49 years, and all spoke Haitian Creole as their primary language. Approximately 94% were covered by the state’s public insurance plan, and approximately half had an active psychiatric diagnosis. Participants joined at any point in the perinatal period, from pregnancy through the first year postpartum. Hospital policy does not collect immigration status, and therefore this was not available for analysis (Section 2.1). To support comparison, Appendix A describes the broader population of Haitian Creole-speaking perinatal patients receiving obstetric care at Boston Medical Center during the study period. Of the 196 HM participants, 91 (46.4%; 95% CI 39.6–53.4%) were administered the RHS-15 at one or more time points. An independent-samples Welch’s *t*-test indicated that women who were not screened attended significantly fewer sessions (M = 2.87; SD = 2.35) than those who were screened (M = 5.44; SD = 3.71, t(145.5) = −5.69, *p* < 0.001, d = −0.83).

#### 3.1.1. Engagement

**Figure 1:**
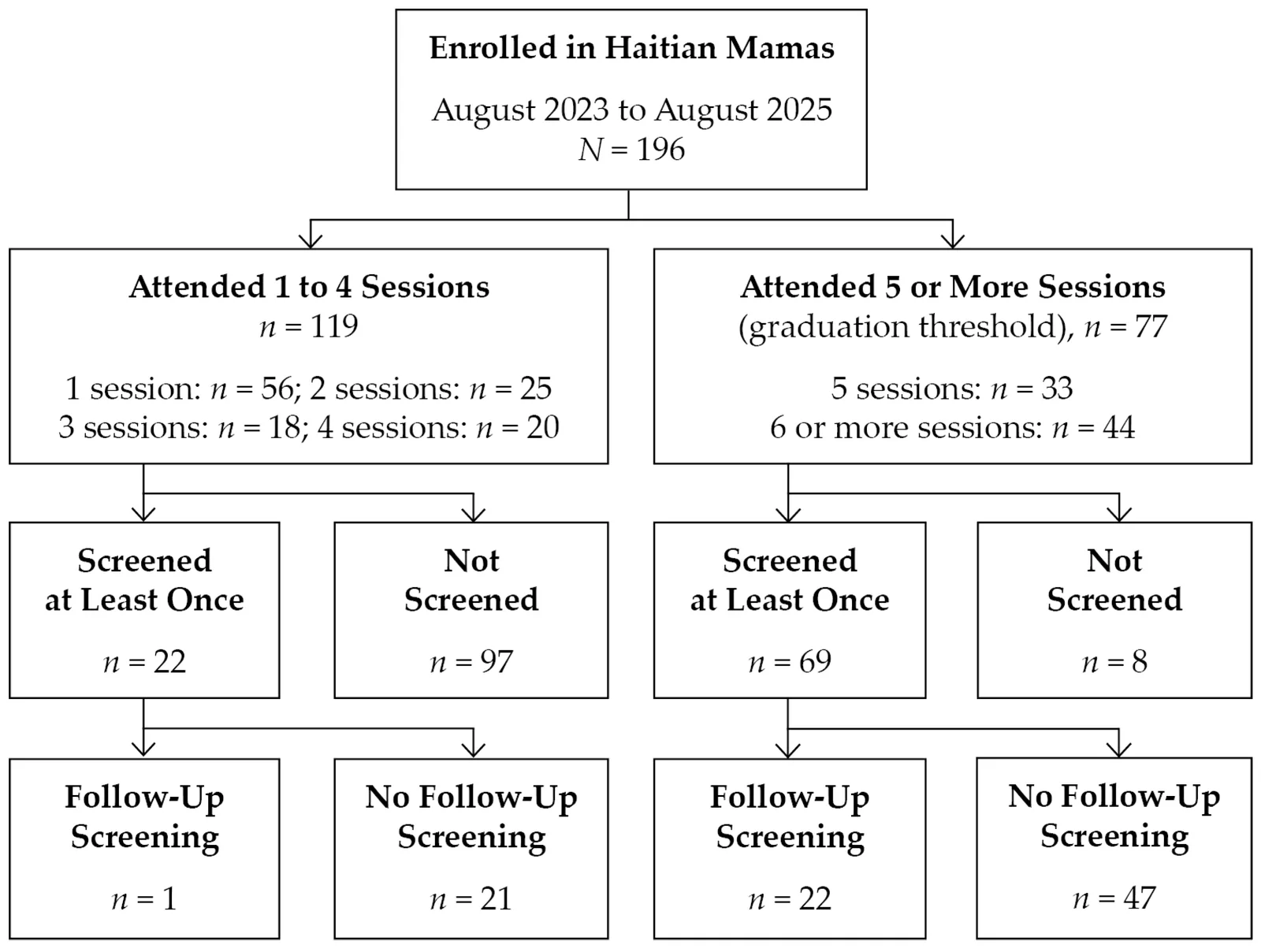
Participant Flow Diagram: Enrollment, Attendance, and RHS-15 Screening. Note: Participants are grouped by whether they reached the five-session graduation threshold. Initial screening occurred between participants’ first and fourth sessions. Follow-up screening was administered at graduation. Of the 23 participants with follow-up screening, 23 had a second screening (T2), four a third (T3), and one a fourth (T4). RHS-15 = Refugee Health Screener-15.

The engagement numbers above should be contextualized within the program’s open access design (Elwy et al., 2020). The five-session model was not adopted until September 2024; before that, patients attended as often or as infrequently as they wanted, needed, or could. The five-session model was adopted after an analysis of attendance in Cycles 1 and 2 showed that participants attended a mean of 3.61 sessions (SD = 3.11; Mdn = 2; IQR = 1–5). A total of 77/196 (39.3%; 95% CI 32.7–46.3%) participants attended at least five sessions. The participant flow diagram (Figure 1) depicts program engagement. Intervention dose varied widely, with participants attending between 1 and 21 sessions; M = 4.05 (SD = 3.30; 95% CI 3.59–4.52), Mdn = 5, IQR = 4–7. The modal number of sessions attended was 1. The mean number of participants per session was 8, exceeding the hospital’s minimum attendance and session-utilization thresholds for group psychotherapy.

#### 3.1.2. Integration of Measurement-Based Care: RHS-15 Screening Administration

**Figure 2:**
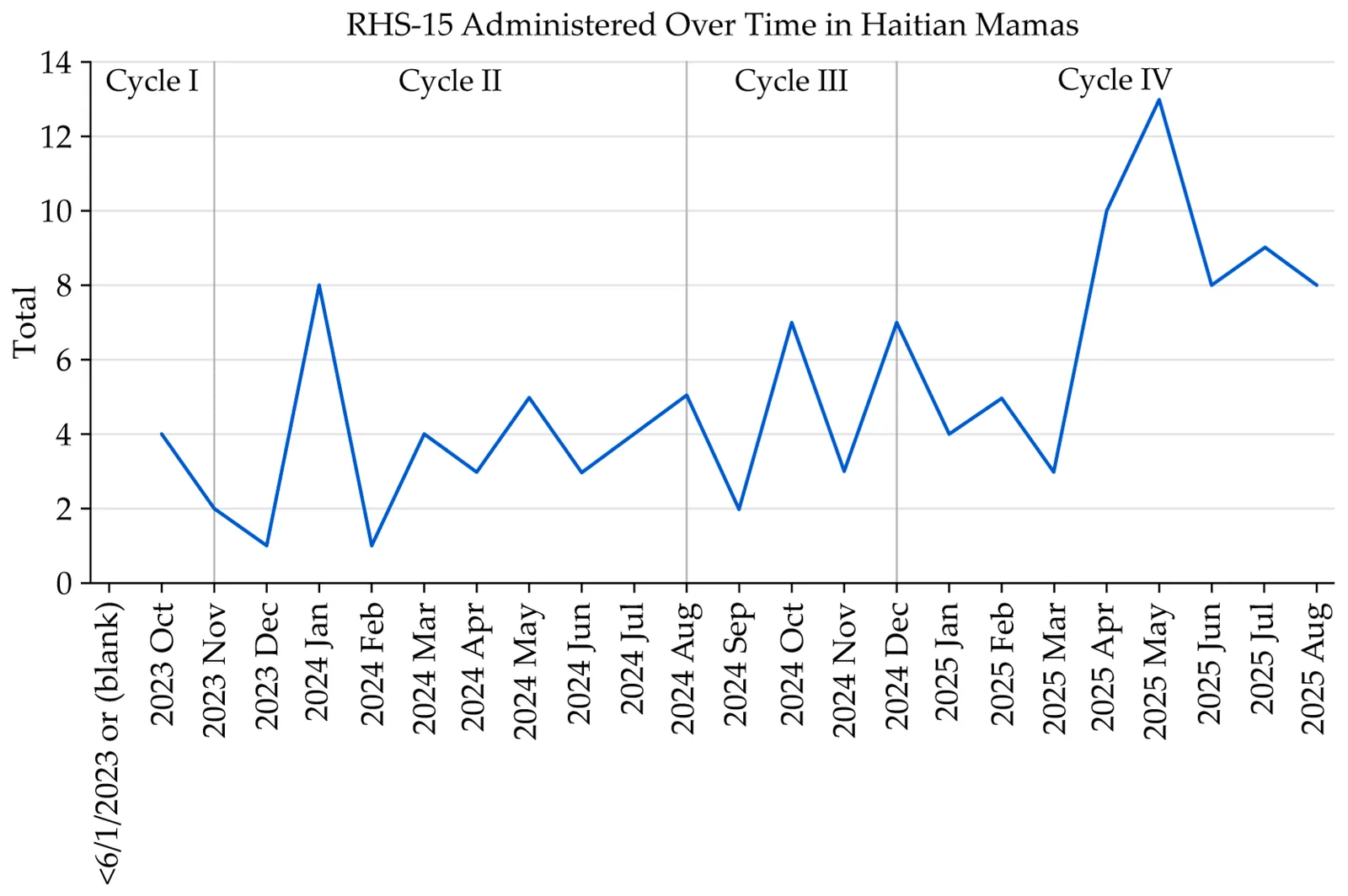
Mental health screening of Haitian Mamas members (August 2023–August 2025).

The 46.4% RHS-15 administration rate reflects a pattern consistent with overall perinatal mental health screening rates at Boston Medical Center, which ranged from 16 to 59% from 2023 to 2025 across language groups (Baul et al., 2026). Overall, screening rates were far below the goal of screening 100% of participants at least once. Of the 196 participants, 91 (46.4%) completed an initial RHS-15 screening (T1), and of these, 23 (25.3%) completed one or more follow-up screenings (T2, n = 23; T3, n = 4; T4, n = 1). However, there was a progressive improvement in measurement fidelity (e.g., 11.5% of participants were screened in Cycle 1 [6/52; 95% CI 5.4–23.0%] vs. 67.4% in Cycle 4 [60/89; 95% CI 57.1–76.3%]), which is itself a finding of the quality improvement process (see Table 2 and Figure 2).

The goals of RHS-15 administration were two-fold: (1) to identify participants with clinically significant distress, such that facilitators could offer a referral to the higher-intensity intervention components of the RWHC BH Care Model, and (2) to determine Haitian Mamas’ capacity to integrate mental health screening into a same-day access group. At initial screening, 70.3% (64/91; 95% CI 60.3–78.7%) of participants had a positive RHS-15 score. In some cases, a positive score was obtained by endorsing a high level of subjective distress (i.e., item 15—distress thermometer) rather than through symptom severity (i.e., items 1–13). Among the minority of participants with follow-up data (n = 23), 65.2% (15/23; 95% CI 44.9–81.2%) screened positive at follow-up. Symptom severity (items 1–13 on the RHS-15) was further investigated using established cut points (Bjärtå et al., 2018). Notably, symptom severity ratings (i.e., Bjärtå et al., 2018) are more precise and are distinct from the positive/negative screening cutoff points (i.e., Hollifield et al., 2013). Both are valid scoring procedures used for different purposes. At initial screening, 43% (39/91) of participants endorsed no clinically significant symptoms, 22% (20/91) mild, 14% (13/91) moderate, and 21% (19/91) severe. This distribution reflects Haitian Mamas’ dual function within the RWHC BH Care Model: a standalone preventive behavioral health intervention or step-down option for participants (59/91) with none to mild symptoms (65%), and an adjunct to higher-intensity behavioral health care for those (32/91) with moderate-severe clinical symptoms (35%). The evaluation of clinical impact, as measured by RHS-15 change scores, is beyond the scope of this manuscript and we make no claims of clinical benefit.

#### 3.1.3. Financial Viability

Group psychotherapy billing was introduced in Cycle 4 (January to August 2025) to test whether billing could offset clinician time (Table 2). Participants with an active psychiatric diagnosis, approximately 50% of participants, were billed for one hour of group psychotherapy, covered under the state’s public insurance plan, which approximately 94% of participants carried. Behavioral health leadership set a prospective benchmark of at least four billable patients per session, the minimum needed to match the Medicaid reimbursement rate for one hour of individual psychotherapy. With an average of eight participants per session, approximately half of whom were billable, typical sessions met this benchmark. Because one postdoctoral psychology fellow and one advanced psychology trainee co-facilitated the group, billing needed to cover only one clinician’s time. While these findings offer preliminary evidence that partial billing can offset clinician time costs, they do not establish overall financial sustainability. A grant supported the program coordinator and other non-billable components, and we did not conduct a full analysis of program costs and revenue.

#### 3.1.4. Adaptability

Four PDSA cycles guided the group’s development across domains of measurement-based care, group structure, and administrative coordination. Table 2 presents the program’s structural evolution across cycles. Table 3 shows how the IWBH, trauma-informed care, and PDSA frameworks jointly shaped four of these decisions.

**Table 2:** PDSA cycle logic: Plan–Do–Study–Act.

Cycle	Plan	Do	Study (Observations)	Act (Adaptations)
Cycle 1 (August–November 2023)	Launch open access group to establish feasibility and maximize initial reach	Open enrollment; no session cap; unlimited attendance; RHS-15 administered by case manager; 288 patient visits; 52 unique patients	Session volume unmanageable without structure; RHS-15 data unreliable (6/52 patients (11.5%) screened); absence of defined endpoint reduced participants’ goal-orientation	Introduced 12-member cap; eight prescheduled/four walk-in slots; cohort graduation ceremonies; RHS-15 transferred to co-facilitators; documentation of clinical notes
Cycle 2 (December 2023–August 2024)	Impose structure; improve measurement reliability; introduce completion milestones	12-member cap (eight prescheduled, four walk-in); prescheduled and walk-in allocation; 6-month postpartum discharge; cohort graduation; co-facilitators administer RHS-15; 479 patient visits; 78 unique patients	Cohort graduation incompatible with open enrollment; graduation at 6 months postpartum relative to OB IBH clinical discharge timeline; data showed participants attended 3.6 sessions on average; co-facilitator screening improved RHS-15 data (34/78 patients (43.6%) screened) but created clinician burden	Replaced time-based discharge (6 mos.) with five-session graduation threshold; appointed program coordinator; shifted to individual graduation ceremonies
Cycle 3 (September–December 2024)	Stabilize operations; maintain pre/post RHS-15 data collection	Five-session graduation threshold; coordinator-administered RHS-15; individual graduation format; 191 patient visits; 46 unique patients	Screening rate maintained with reduced co-facilitator burden (19/46 patients (41.3%) screened); coordinator role essential to operational stability; individual graduation reduced scheduling-related attrition	Retained all Cycle 3 structures; introduced selective billing to test group sustainability; RHS-15 screening support provided to program coordinator
Cycle 4 (January–August 2025)	Test billing integration without compromising access or attendance	Selective billing implemented; coordinator role continued; full Cycle 3 structure retained; 522 patient visits; 89 unique patients	Billing did not compromise attendance or access; coordinator role remained essential; pre/post RHS-15 screening improving (60/89 patients (67.4%) screened)	Current program state.

Note: OB IBH = Integrated Behavioral Health Team embedded in the OBGYN department. Screening counts are reported per cycle. Participants who attended in more than one cycle are counted in each cycle in which they were screened; across the study period, 119 screenings were completed by 91 unique participants.

**Table 3:** How the guiding frameworks informed program decisions.

Program Decision	PDSA: What the Data Showed and What Changed	IWBH: Integration Within OB/GYN	TIC: Trauma-Informed Care Principles
Prescheduled and same-day slots (Cycles 1 to 2)	Cycle 1 session volume was unmanageable, and insurance-covered transport could not be arranged for walk-in participants without fixed travel dates. The team introduced a 12-member cap with eight prescheduled and four same-day slots.	Reserving same-day slots preserved warm hand-offs from OB/GYN providers during that day’s clinic.	Patient feedback about transport shaped the change (collaboration and mutuality). Keeping same-day access preserved patients’ choice in how they joined (empowerment, voice, and choice).
Five-session individual graduation (Cycles 2 to 3)	Cycle 2 data showed participants attended about four sessions on average. Cohort graduation conflicted with open enrollment. The team replaced the 6-month postpartum discharge with a five-session threshold and individual ceremonies.	The new endpoint replaced a discharge rule tied to the OB IBH team’s clinical timeline with one that fit group participation.	Individual ceremonies, with a certificate and an invitation to share, recognized each mother’s experience (empowerment, voice, and choice). Graduation reflections gathered feedback for the next cycle (collaboration and mutuality).
In-person Haitian Creole interpretation (Cycles 1 to 4)	Field notes documented repeated sessions without an interpreter, which prevented co-facilitation. Consistent in-person interpretation became a target for adaptation.	The team met with interpreter services and a separate hospital department to understand their constraints and plan coverage, leading to more consistent in-person interpretation.	In-person, culturally concordant interpretation addressed cultural and linguistic needs (cultural, historical, and gender issues). Working directly with interpreter services reflected trustworthiness and transparency.
Front-desk check-in for same-day participants	OB/GYN administration requested that same-day participants check in at the front desk before joining the group. The team changed its workflow to meet this request.	Aligning with OB/GYN administrative workflow kept the group integrated within the department’s operations.	Responding to clinic partners’ preferences reflected collaboration and mutuality, while maintaining same-day patient access.

Note: PDSA = Plan–Do–Study–Act; IWBH = Integrated Women’s Behavioral Health; TIC = trauma-informed care, using the six principles of the Substance Abuse and Mental Health Services Administration (SAMHSA). OB IBH = Integrated Behavioral Health Team embedded in the OB/GYN department.

### 3.2. Qualitative Findings: Provider-Perceived Acceptability

Over two years, four providers contributed to the provider reflections. The reflections addressed providers’ experiences facilitating the group, adapting the group, and the group content. Challenges Arising for Providers was the most prominent theme, accounting for the largest share of coded excerpts and recurring across all four PDSA cycles. The remaining four themes were represented at similar levels. The current paper presents provider findings (see Table 4), while patient-related feedback is discussed in a companion manuscript (Sokoto et al., under review).

#### 3.2.1. Interdisciplinary Collaboration

The Haitian Mamas group could not have functioned without the active participation of professionals well beyond the two primary co-facilitators. For example, language interpreters, case managers, midwives, nurse practitioners, OB/GYN physicians, the RWHC program coordinator, trainees from multiple disciplines, clinic volunteers, and community contributors all played documented roles in supporting the group.

“Teamwork was key this session: psychology intern assisted with clothing distribution and organization of resources, [staff member] assisted in writing a pt [patient] letter, [staff member] handled transport and [staff member] assisted in transport when time arose…; [staff members] also sorted out clothing, [staff member] helped, etc. It was a more hands-on week than usual.”

Community contributions, including a Haitian chef providing food at subsidized cost, extended the program’s reach beyond what institutional and grant budgets could have supported. Additionally, group leaders described the program’s training function, in line with the IWBH framework, as a growing and valued component.

As one facilitator noted,

“Our group has a growing arm in training. Many trainees have joined our group to shadow and see the work that is being done. New employees too have asked to shadow and have been welcomed.”

#### 3.2.2. Challenges Arising for Providers

The group facilitator’s field notes documented sustained operational, institutional, and relational challenges across all four PDSA cycles (Suphanchaimat et al., 2015). Recurring challenges included financial sustainability, group structure and logistics, managing facilitator absences, advocating for consistent in-person interpretation, and transportation barriers. In Cycle 1, when patients would walk in to the group and there was no coordinator to support prescheduling, insurance-covered transportation (Patient Transport (PT-1)) to the hospital was challenging to obtain:

“Ride concern was brought up with scheduling [transport], but if patients do not have fixed dates to return, they can only wait until Friday beforehand (we re-schedule no shows on the following weeks, so if a pt [patient] is supposed to come 2–3 weeks later, it can be 4–5 weeks later depending on how many drop-ins and no shows were there in previous weeks).”

On three consecutive occasions in one period, no interpreter was available, requiring the Haitian Creole-speaking co-facilitator to interpret simultaneously, a role that prevented co-facilitation and placed her in an untenable professional position.

“We had no interpreter. [The Haitian Creole-speaking co-facilitator] had to interpret, making co-facilitation for [the other co-facilitator] impossible…”

Early in implementation, the drop-in format also strained staff capacity, which informed the attendance cap introduced after Cycle 1 (Table 2):

“Having too many people in the group—this is probably a health hazard with many mothers and babies in one room. It is also stretching staff… Resources are limited [and] the drop-in group model requires admin.”

#### 3.2.3. Group Development and Adaptations

A defining feature of the program was its responsiveness, through which facilitators could adapt group content to the social–political context (Elwy et al., 2020). For example, with changing US immigration policies, providers allowed time for processing immigration policy-related concerns in sessions (e.g., how to obtain a passport for a newborn using the United States Postal Service website):

“We [providers] spent the entire hour detailing the process [The USPS website instructions were in English). [We] set a goal of having the process of obtaining a passport [for your newborn child] translated [to Haitian Creole].”

Additionally, the group documented and evaluated decisions about attendance caps, graduation thresholds, and scheduling logistics.

For example, when the five-session format was introduced, facilitators adapted enrollment to provide mothers with flexibility:

“We had 6 mothers left from the previous cycle who are due to graduate… We opted to invite them into the new cycle… We have new punch cards, and mums can schedule their 5 sessions according to availability. Mums were receptive to the idea.”

#### 3.2.4. Provider Emotional Response

Facilitator field notes offered a candid account of the emotional experience of facilitating this group, including grief and compassion. One Haitian provider wrote the following:

“It was hard as a Haitian provider to sit through the hard conversation about Haiti… and not get lost in thoughts and emotions.”

Moral distress was documented across multiple field notes. Facilitators described feeling helpless with participants’ immigration questions and sessions feeling heavy when housing needs exceeded the group’s scope:

“I felt moved that the member felt so guilty for being tired raising her baby alone… It makes me sad that she’s so alone.”

Field notes also captured positive emotional responses, as providers noticed and affirmed members’ strengths:

“Mothers… really wanted to share the things they feel they are good at. I really liked to hear them speak about themselves in affirmative ways.”

#### 3.2.5. Provider and Patient Partnership

One of the most distinctive features of the group was how much participants co-shaped it over time (Elwy et al., 2020). Facilitators responded in real time to participant needs that emerged unexpectedly in sessions.

“We had planned a subject topic, but it turned out many (about four) women had given birth recently. We then recognized each baby. We also opened up the session for mothers to process their birthing experience.”

Members also shaped the group’s structure. Before the five-session format was introduced, facilitators asked members for their input:

“Shared decision making regarding upcoming changes to group format. Members[’] feedback was elicited on the 5 sessions format. Group members reported they would prefer to spread out sessions because of the weather and the distance from the hospital. Others preferred having frequent sessions.”

Facilitators actively solicited participant feedback at graduation and documented responses in field notes. Patient-focused acceptability is further discussed in a separate manuscript (Sokoto et al., under review).

## 4. Discussion

Haitian Mamas was developed in response to the need for accessible, preventive, and culturally responsive behavioral health services for perinatal patients with forced migration histories. The findings from this quality improvement study suggest that a same-day access, OB/GYN-based preventive behavioral health group in a safety-net hospital is operationally feasible.

### 4.1. Feasibility: Reach, Engagement, Screening Integration, and Adaptability

Over two years, 196 women enrolled in Haitian Mamas. Although behavioral health screening increased across PDSA cycles, from 11.5% in Cycle 1 to 67.4% in Cycle 4, especially after we added a program coordinator, screening still fell short of our goal of screening every participant. The screened subgroup may not represent the 105 of 196 participants who were not screened, who attended significantly fewer sessions. Administering a culturally validated screener, with culturally responsive behavioral health providers available within OB/GYN, addressed some of the cultural validity concerns and training limits on behavioral health screening in traditional OB/GYN settings (Itani et al., 2025; Poleshuck & Woods, 2014). For screened patients, HM was able to provide low-intensity care and refer appropriate cases for higher-intensity care. Preventive interventions are one way of enhancing screening and access to behavioral health care for perinatal patients.

Engagement met the feasibility benchmark, with sessions averaging eight participants, above the institution’s minimum of four. This finding matters given Black and brown migrant populations are documented to have among the lowest rates of behavioral health utilization, driven by multiple structural barriers (Bauldry & Szaflarski, 2017; Derr, 2016). Haitian Mamas may have addressed some of these barriers through same-day access, child minding, in-person interpretation, group modality, and trauma-informed culturally responsive care (Markey et al., 2024). Behavioral health interventions for perinatal forced migrant populations in safety-net hospitals need to address the social determinants of health tied to immigration.

Adaptability is reflected in the structural changes documented across the four PDSA cycles, which were associated with more consistent screening, reduced provider burden, and closer alignment with available clinical resources. Provider field notes show that patients helped shape the group’s format and content. It is also possible that the facilitators’ identity as trainees shaped the development and implementation of Haitian Mamas. The most significant change was adding a program coordinator in Cycle 3 to manage referrals, scheduling, and supplies, which reduced provider burden and supported consistent screening and documentation (Myors et al., 2013).

### 4.2. Provider-Perceived Acceptability: Facilitators and Barriers

Provider-perceived acceptability was assessed through a thematic analysis of provider field notes. These findings reflect provider perspectives, not patient-reported outcomes. Provider field notes indicated that interdisciplinary collaboration was key to Haitian Mamas’ operational feasibility. This finding highlights the importance of shared purpose, role flexibility, and trust in interprofessional perinatal collaboration (Keedle et al., 2023). For example, an OBGYN provider reaching out to the BH team led to a needs assessment for BH services that resulted in the development and implementation of Haitian Mamas. This relationship may have shaped the quality improvement project’s trajectory. The OB team welcomed RWHC BH providers and provided feedback indicating that they were making a significant contribution to OB. The necessity of interdisciplinary collaboration highlighted in our results aligns with the Integrated Women’s Behavioral Health framework (Crawford et al., 2022) and suggests practical implications for resource distribution through collaboration. Integrative behavioral health models have historically addressed behavioral health needs in specialty clinics (Caudle & Khatri, 2024). Although most are appointment-based with individual care models, this study suggests a same-day access, OBGYN-based group care modality as an extension of IBH for perinatal patients (Carroll et al., 2020; Yeshitila et al., 2024).

Providers reported that using existing infrastructure facilitated the implementation and continuity of care for Haitian Mamas (Novick et al., 2015; Webb et al., 2023). Health systems seeking to replicate this model would ideally have access to the following: (a) a behavioral health champion within the OB/GYN clinic; (b) co-located space within or adjacent to an obstetric or women’s health clinic; (c) institutional group programming infrastructure or grant funding for a dedicated program coordinator; (d) access to in-person medical interpreter services (if group leaders are not linguistically concordant); (e) child-minders; and (f) culturally responsive behavioral health co-facilitators trained in perinatal mental health. This finding matters because not all hospitals have the same resources. Alternative resources could be considered, such as telehealth groups, where space, transportation access, or child-minding availability is limited.

Our findings on the barriers to implementing the group across different points of the PDSA are established barriers to care for forced migrants in high-income countries, including patient access to transportation and unreliable language translation (Markey et al., 2024). Providers also identified moral distress stemming from systemic constraints, which aligns with the literature on providers working with migrant populations and in safety-net hospitals (Suphanchaimat et al., 2015). Evidence shows that systemic acknowledgment of this distress makes a difference for providers (Vinckers & Landeweer, 2025). Health systems must provide adequate support and resources (i.e., co-facilitation, consistent supervision and training) for clinical staff working with high-need populations like forced migrants.

### 4.3. Sustainability: Financial Viability

Haitian Mamas operated continuously for two years across four PDSA cycles. While the billing findings offer preliminary evidence that partial billing can offset the cost of one clinician’s time, the fact that typical sessions met the benchmark of four billable patients does not demonstrate overall financial sustainability. In our setting, the program coordinator, a non-clinical role that proved essential to consistent screening, was funded by the ReMHI grant, as were other non-billable components. Sites without outside funding would need existing administrative infrastructure to support these components, and the current data cannot determine whether the program can continue after the grant ends.

### 4.4. Limitations

Several limitations of this quality improvement study are worth acknowledging. First, as a single-site quality improvement study, the generalizability of the Haitian Mamas group to other settings and populations requires further evaluation. Of note, the mission of Boston Medical Center is to serve the underrepresented, using teaching hospital resources such as in-person interpretation services and medical student volunteers as potential child minders. This context must be considered when implementing the group at institutions that may not offer such support. Second, financial sustainability should be interpreted cautiously, as the data presented here cannot assess the Haitian Mamas group’s viability after the ReMHI grant ends. Third, two analysts had dual roles: independent auditing by two analysts without a facilitation role (JTT, RG) was incorporated to reduce interpretive bias, though the facilitating authors remained involved in theme development and interpretation, a common constraint in practitioner-embedded quality improvement work of this kind. Finally, we collected provider field notes during intervention development and initial implementation. Longitudinal research is needed to understand the long-term impacts of group-based interventions for perinatal forced migrants (Kirsch et al., 2024).

### 4.5. Future Directions

Building on the implementation experience described here, future work will test the same-day access, OB/GYN-based preventive behavioral health pathway as an implementation strategy for an evidence-based intervention for perinatal populations with trauma histories. This will generate the preliminary mental health and obstetrics outcome and utilization data needed to support a cost-utility analysis (Sanders et al., 2016) and a controlled effectiveness trial (Hariton & Locascio, 2018).

## 5. Conclusions

Our findings from Haitian Mamas suggest that a same-day access, OB/GYN-based preventive behavioral health group is operationally feasible in a safety-net hospital setting. Informed by the IWBH framework, SAMHSA’s trauma-informed care principles, and the PDSA framework, this paper provides an example of developing and implementing such a group for perinatal forced migrant women.

## Figures and Tables

**Table 1:** Haitian Mamas cross-departmental and interdisciplinary providers.

Role	Qualifications	Function
Co-facilitators	Doctoral-level clinical and counseling psychology (predoctoral or postdoctoral); at least one facilitator of migrant identity and racially/ethnically concordant to group members; completed six-session cross-training in perinatal mental health at program launch; weekly supervision from licensed psychologists with expertise in immigrant mental health and perinatal mental health.	Co-facilitated using dual-role model: one provider maintained group process, and one attended to individual clinical needs. Providers regulated with each other during high-emotion-content sessions. RHS-15 administration.
Haitian Creole interpreter	Culturally self-identified as Haitian; attended every session in person.	Linguistic and cultural interpretation throughout all sessions. Cultural expert.
Program coordinator	Master’s-level social work professional. Trained in program protocol and population health.	Electronic Health Record referral management; pre-session scheduling and outreach; child-minding coordination; interpreter scheduling; supply sourcing; OB/GYN liaison; RHS-15 measurement coordination; program documentation and grant management.
Child-minding volunteers	Medical students; hospital volunteers; received basic program orientation before participating and could opt out of child-minding.	On-site childcare in adjacent space during every session, enabling attendance without independent childcare arrangements.
Interdisciplinary trainees	Predoctoral psychology interns, public health graduate students, social work volunteers.	Observation for clinical training in group psychotherapy with forced migrant populations.
OB/GYN referral network	OB/GYN physicians, nurse practitioners, midwives, case managers, and behavioral health providers, including psychiatrists and therapists.	Participant identification and referral or warm hand-offs (transfer in real time from OB provider into the group); targeted content presentations on reproductive health (midwives); post-group care coordination (case managers).

**Table 4:** Provider themes: superordinate themes, subordinate themes, and definitions.

Superordinate Theme	Subordinate Themes	Definition
Interdisciplinary Collaboration	Importance of coordination; trainee integration; volunteer and coordinator support; wider network contributions	Collaboration across professional disciplines that facilitated group delivery.
Provider and Patient Partnership	Members inform group components; members inform discussion topics	Bidirectional feedback with patients was solicited and observed, and it impacted group formation.
Group Development and Adaptations	External resources and guest speakers; format decisions; facilitator adjustments; group content; graduation and endings	Documented changes to group content, format, and structure made in response to participant needs and operational learning.
Challenges Arising for Providers	Finances; group membership duration; transportation; group facilitation; language translation	Operational-, institutional-, and contextual-level challenges encountered during group program delivery.
Provider Emotional Response	Moral distress and shared grief; provider burden; positive strengths identification	How facilitators and staff were emotionally affected by group content and by the experience of providing care to this population.

## Data Availability

Qualitative data and analysis files are stored securely on the Boston Medical Center institutional drive and are available from the corresponding author upon request with appropriate data use agreements and IRB approval.

## Abbreviations

The following abbreviations are used in this manuscript:

BH	Behavioral health
DSM-5	Diagnostic and Statistical Manual of Mental Disorders, Fifth Edition
HM	Haitian Mama
IWBH	Integrated Women’s Behavioral Health
LICSW	Licensed independent clinical social worker
OB/GYN	Obstetrics and Gynecology
OB IBH	Obstetrics Integrated Behavioral Health
PDSA	Plan–Do–Study–Act
PMH	Perinatal mental health
PTSD	Post-traumatic stress disorder
ReMHI	Refugee Mental Health Initiative
RHS-15	Refugee Health Screener-15
RWHC	Refugee Women’s Health Clinic
RWHC BH	Refugee Women’s Health Clinic Behavioral Health
SAMHSA	Substance Abuse and Mental Health Services Administration
TIC	Trauma-informed care
TIDieR	Template for Intervention Description and Replication
US	United States

## Appendix A. Demographic Characteristics of the OB/GYN Patient Population

**Table A1:** Education, marital status, and parity of Haitian Creole-speaking perinatal patients receiving obstetric care, 2023 to 2025.

Characteristic	%
Education	
No schooling	0.6
8th grade or less	14.2
Some high school	18.9
High school degree	39.6
Some post-secondary or vocational training	5.1
College graduate	9.9
Not reported	11.7
Marital status	
Married	41.1
Unmarried with a significant other	9.8
Single	46.6
Divorced or unspecified	2.5
Parity (current pregnancy)	
First	74.0
Second	20.6
Third	4.1
Fourth	1.2
Fifth	0.1

Note: *N* = 1425. Data describe the broader population of Haitian Creole-speaking perinatal patients receiving obstetric care at Boston Medical Center during the study period, not Haitian Mamas participants only. Percentages for “Not reported” (education) and “Divorced or unspecified” (marital status) were calculated as the remainder of reported categories. Parity reflects the pregnancy for which patients were receiving obstetric care. Immigration status is not collected or documented per hospital policy and is therefore not available for analysis.

## Appendix B. Sample Provider Initial Codes

**Table A2:** Sample of provider initial codes by clinician and common themes, field-note pages 2 to 5.

Page	Clinician 1 Initial Codes	Clinician 2 Initial Codes	Common Theme
2	Comment on food and music again	Food and music planning	Food and music discussed
	Interpreter mentioned	Interpreter requesting concerns	Interpreter request discussed
	—	Psychoeducation on dissociation	—
	Primary staff and others, such as case managers, RWHC supervisors, and midwives, mentioned	Multidisciplinary team	Multiple providers (multidiscipline)
	Mention this again: food and music said by patients?; other provider feedback	Highlighting purpose of group; providers ask patients for experience feedback	Providers ask patients for group-related feedback
3	Issue with medical software and provider	Logistical concerns in OB setting	Logistical concern with OB workflow and registration
	—	Interpreter challenges	—
	Money question arises (relates to food cost)	Food sustainability	Food cost discussion
	Transportation	Transport challenges	Transportation challenge
	Noticing attendance	Expertise able to identify clinical presentations	—
	Use case managers	Multidisciplinary: case managers and interpreters	Use of case managers
	—	Good experience with interpreter	—
4	Provider has personal immigration concern; group cancellation: possible disruption?	—	—
5	Provider interest in learning members’ language; various topics being discussed each session	Asking members questions: What keeps them coming to the group?	Providers ask patients what attracts them to group sessions

Note: Two clinicians, the lead analysts, independently coded each page of the provider field-note document. A common theme was recorded when both clinicians’ initial codes for the same section of text were similar; a dash indicates no corresponding code or common theme. Page numbers refer to pages of the field-note document. OB = obstetrics; RWHC = Refugee Women’s Health Clinic.

## Appendix C. Sample Provider Initial Coding and Themes

**Table A3:** Sample of provider initial codes by clinician and resulting common themes.

Common Theme	Clinician 1 Initial Code	Clinician 2 Initial Code
Food and music discussed	Group elements discussed (food/music)	Planning for food and music
Interpreter request discussed	Interpreter/group aid	Coordinating in-person interpreter
Multiple providers (multidiscipline)	Group professional support	Multiple providers and disciplines
Providers ask patients for group-related feedback	Patient and provider feedback/work together	Seeking patient feedback
Logistical concern with OB workflow and registration	Hospital problems identified: patient registration issues	Challenges with OB workflow
Food cost discussion	Cost issue (food)	Challenges with food cost
Transportation challenge	Challenge to patient care: transport to group	Transportation challenge
Use of case managers	Professional support: case managers	Case manager support
Providers ask patients what attracts them to group sessions	Provider and patient feedback (attracts to group)	Seeking patient feedback
Group numbers rose and discussion about procedures	Group issue: large numbers	Increased group numbers and logistics discussion
Question limit for ongoing goodbye gifts for leaving group members	Group issue: funding for patient gifts discussed	Discussing sustainability of graduation gifts
Transportation as a patient stressor due to limited resources	Issue: transportation limitations for patients	Patient transportation and limited resources
Food cost discussed	Financial cost for food	Food cost concern
Volunteer support added	Professional support: volunteers	Support need for volunteers
Revise allowed time frame for group participation (6 months)	Group issue: members’ time frame adjusted	Change group membership limit
Provider feeling guilt due to gap in group attendance time	Provider guilt about members’ wait for group	—
Collective concern felt about patients’ loved ones in Haiti	Patients and providers share concerns	Collective concern for the state of Haiti
Transportation: PT-1 scheduling	Transport is a focus for providers	PT-1 transport scheduling challenges
Haitian provider had empathy with patients for events in Haiti	Provider shares emotions about events in Haiti	Provider empathy for Haiti
Added other elements to group, such as gifts and certificates for members	Add gifts to group	Gifts and certificates at graduation
Photos at graduation (closure)	Add photos to group	Photo session at graduation
Gifts and certificates helpful (tangible transition item)	Gifts and certificates helpful	Gifts and certificates at graduation
Heavy feeling for provider: inability to help with patient resources such as housing	Provider heaviness: cannot help with housing	Provider helplessness on housing
Group number increases	Patient numbers rise	Group number increase
Patient transportation expiring is a challenge	Patient transport challenge	Transport assistance expiration
Information packet with patient resources	Information packet (helpful)	Provide packet with resources
Haitian provider needs to interpret; leading and translating is challenging	Provider doing two jobs: feels challenged	Haitian provider needing to interpret
Interpretation issue	Interpretation issue	Interpreter challenge
Case management in group on topics (e.g., child abuse and DCF, SNAP benefits)	Case manager help: professional support	Case management–related topics
Teamwork of a large team of providers; multidisciplinary help	Professional support: different providers	Multidisciplinary team support

Note: Coding question: How did providers feel about offering the group? Two clinicians, the lead analysts, independently coded the same field-note pages. Each common theme reflects initial codes shared by both clinicians. These common themes were then grouped into the Phase 1 themes shown in Appendix D. In the original coding spreadsheet, each clinician also color-coded initial codes into broader categories. Clinician 1: group content and resources, provider supported by other providers, provider emotions and experience, patient and provider empathy and patient feedback, community support, and group issues and challenges. Clinician 2: multidisciplinary teamwork, system-level workflow, patient feedback, adapting group structure, impact on provider emotions, and training as support. A dash indicates that no code was recorded. OB = obstetrics; PT-1 = insurance-covered patient transportation; DCF = Department of Children and Families; SNAP = Supplemental Nutrition Assistance Program.

## Appendix D. Themes and Subordinate Themes Before and After Peer Expert Review

**Table A4:** Evolution of provider themes and subordinate themes across three rounds of analysis and peer expert review.

Superordinate Theme	Phase 1: Initial Themes	Phase 2: After Auditors’ Written Review	Phase 3: After Auditor Consultation	Final Themes
Multi-discipline provider support **→** Interdisciplinary Collaboration	Importance of coordination between multiple care providers during sessions and for follow-up Training new team members and roles of trainees Immigration-related resources shared Community members’ contributions	Importance of coordination between multiple care providers Training new team members and roles of trainees Immigration-related resources shared Community members’ contributions	Importance of coordination between multiple care providers Training new team members and roles of trainees Immigration-related resources shared Wider network contributions (support from within the hospital and from the community beyond the hospital system)	Importance of coordination Trainee integration Volunteer and coordinator support Wider network contributions
Challenges Arising for Providers	Budget and finance concerns Session length and timing revised Transportation challenges Group leader concerns Advocating for translation and interpretation Group structure Limited immigration-related materials; challenges finding immigration support	Budget and finance concerns Session length and timing revised Transportation challenges Group leader concerns Advocating for translation and interpretation Issues related to group structure Limited immigration-related materials; challenges finding immigration support	Budget and food finance concerns Session length and timing revised Transportation Group leader concerns Advocating for translation and interpretation Issues related to group structure Limited immigration-related materials (challenges finding immigration support)	Finances Group membership duration Transportation Group facilitation Language translation
Group planning process and adaptations → Group Development and Adaptations	Resources shared during group Group structure Leader Questions about change Emotions	Resources shared during group Group structure layout change Leader adjustments Group topic New element of group added	External resources and guest speakers Group structure layout change Leader adjustments Group topic Graduation and group endings	External resources and guest speakers Format decisions Facilitator adjustments Group content Graduation and endings
Provider Emotional Response	Provider shared emotions with patients Negative feelings related to challenging patient experiences	Positive feelings from group empowerment Provider empathetic countertransference emotions Negative feelings related to challenging patient experiences	Positive strengths identification Moral distress and shared grief Provider burden	Positive strengths identification Moral distress and shared grief Provider burden
Provider and Patient Partnership	Group members share concrete elements of group they enjoyed Members inform provider process on group topics and structure	Group members share concrete elements of group they enjoyed Members inform provider process on group topics and structure Group structure	Group members share concrete elements of group they enjoyed Members inform provider process on group topics and structure Group structure	Members inform group components Members inform discussion topics

Note: Phase 1 grouped the codes shared by both analysts (Appendix B) into initial superordinate themes and subordinate themes. In Phase 2, the two peer expert auditors reviewed the coding spreadsheet and provided written feedback to clarify themes. In Phase 3, the primary analysts met with the auditors to further refine themes. All co-authors confirmed the final theme and subordinate theme names reported in Table 4. Arrows indicate superordinate themes renamed in the final stage.
